# Preventing inpatient falls with injuries using integrative machine learning prediction: a cohort study

**DOI:** 10.1038/s41746-019-0200-3

**Published:** 2019-12-12

**Authors:** Lin Wang, Zhong Xue, Chika F. Ezeana, Mamta Puppala, Shenyi Chen, Rebecca L. Danforth, Xiaohui Yu, Tiancheng He, Mark L. Vassallo, Stephen T. C. Wong

**Affiliations:** 1 Bioinformatics and Biostatistics Cores and Systems Medicine and Bioengineering, Houston Methodist Cancer Center, Houston, TX 77030 USA; 2 Department of Informatics Development, Houston Methodist Hospital, Houston, TX 77030 USA; 3 Department of Quality Operations, Houston Methodist Hospital, Houston, TX 77030 USA

**Keywords:** Disease prevention, Risk factors

## Abstract

Patient falls during hospitalization can lead to severe injuries and remain one of the most vexing patient-safety problems facing hospitals. They lead to increased medical care costs, lengthened hospital stays, more litigation, and even death. Existing methods and technology to address this problem mostly focus on stratifying inpatients at risk, without predicting fall severity or injuries. Here, a retrospective cohort study was designed and performed to predict the severity of inpatient falls, based on a machine learning classifier integrating multi-view ensemble learning and model-based missing data imputation method. As input, over two thousand inpatient fall patients’ demographic characteristics, diagnoses, procedural data, and bone density measurements were retrieved from the HMH clinical data warehouse from two separate time periods. The predictive classifier developed based on multi-view ensemble learning with missing values (MELMV) outperformed other three baseline models; achieved a cross-validated AUC of 0.713 (95% CI, 0.701–0.725), an AUC of 0.808 (95% CI, 0.740–0.876) on the separate testing set. Our studies show the efficacy of integrative machine-learning based classifier model in dealing with multi-source patient data, which in this case delivers robust predictive performance on the severity of patient falls. The severe fall index provided by the MELMV classifier is calculated to identify inpatients who are at risk of having severe injuries if they fall, thus triggering additional steps of intervention to prevent a harmful fall, beyond the standard-of-care procedure for all high-risk fall patients.

## Introduction

A fall is defined as “an untoward event which results in the patient coming to rest unintentionally on the ground or other lower surface”.^1^ Predicting falls, especially the potential severity of a fall, is quite tough because, based on Morse’s classification, there are several types of falls, including accidental, anticipated physiological, and unanticipated physiological falls.^2^ Some classifications have further created behavioral (intentional) and assisted fall types in addition to these classes.^3^ Patient falls during hospitalization are serious and costly. Given the already compromised state of individuals who are in hospital settings, falls often lead to other complications, such as fractures, lacerations, and/or significant internal bleeding. In the United States alone, thousands of patients fall in hospitals every year, with about 30–50% resulting in injury.^4–6^ Often, patients injured from falls require additional treatment and extended hospital stays. Accidental patient falls complicate an estimated 2% of hospital stays, and the rates of falls range from 3.3 to 11.5 falls per 1000 patient days. In a study performed at three midwestern hospitals, fall injuries added 6.3 days to hospital stays. On average, the cost implication for a fall with injury is around $14,000.^7–9^ Between 2009 and 2015, The Joint Commission’s Sentinel Event database compiled a total of 465 severe falls occurring in hospitals. 63% of these severe falls resulted in death, while the remaining patients sustained serious injuries.^10^

While patient falls can be related to the type of care setting, age, mental status, illness, medication, and others, hospitals strive to prevent and keep fall incidents to the barest minimum. To prevent patient falls, screening tools are implemented. Fall risk assessments are performed at the patient bedside to identify who is at the highest risk upon hospitalization, and standardized practices are adopted, especially for patients with high fall risk. This type of assessment employs specific screening instruments, with the most prevalent being the Morse Fall Scale, St. Thomas Risk Assessment Tool in Falling elderly inpatients (STRATIFY), Resident Assessment Instrument (RAI), Fall Risk Assessment Tool, Hendrich Fall Risk Model, High Risk for Falls Assessment Form, Royal Melbourne Hospital Risk Assessment, and Hester Davis scale for fall risk assessment.^11–16^ These tools are used to identify patients who are likely to fall based on their intrinsic or medical characteristics, e.g., psychological status, mobility dysfunction, fall history, elimination frequency/dependence, acute/chronic illnesses, medications taken, and sensory deficits. These instruments are commonly utilized by nurses upon patient admission and are periodically updated, e.g., per shift, daily, or weekly, depending on the acuity level of the patient.

Although a variety of adequate screening tools are available, further research is needed in several areas. For example, most existing tools are not designed to predict whether a fall would be severe and thus are of little help in their application for preventing severe injuries and other consequences. Predicting the severity of patient falls in at risk patients offers the opportunity to identify medical and physiological factors that can help healthcare practitioners to offer more tailored interventions.

The objective of this study is to develop a general predictive model for severity of falls among patient populations, using an advanced machine learning method multi-view ensemble leaning to efficiently exploit the multidimensional patient data.^17,18^ Our goal is to provide an automatic severity index to predict if a fall will be severe in patients with an appreciable fall risk so that appropriate interventions and more attention can be provided to these patients and prevent such accidents from happening.

## Results

### Study design

There were 1837 fall incidents occurring among 1692 patients in the training set, of which 297 cases (16.2%) were severe fall incidents. Testing was performed on data from the period of May 2016 to December 2016, including 306 fall incidents (275 patients), of which 33 cases (11%) were severe fall incidents. For each fall case, we collected data including patient demographics like age, race, sex, disease diagnosis, bone density measures if available, and procedural data if available. There are 3170 distinct ICD codes in 723 disease category recorded in the training set, and 1891 distinct ICD codes in 442 disease categories in the testing set. About 5% of patients had forearm type bone density measures (92 cases) and 7.5% of patients had dual femur type bone density measures (137 cases). About 30.4% (559 cases) of fall incidents had a procedure performed within 10 days before the patient fell, with 202 distinct procedure codes recorded. In the testing set 31.7% (97 cases) had procedures with 39 distinct procedure codes recorded. A summary of the training set and testing set is provided in Table 1.

**Table 1:** Characteristics and variables of the 1837 training data and 306 testing data.

Characteristics	Training set (*n* = 1837)	Testing set (*n* = 306)
No. Unique Patients	1692	275
Age, Mean (SD)	63.0 (15.7)	64.6 (15.8)
Sex, No. (%)		
Female	942 (51.3)	148 (48.4)
Male	895 (48.7)	158 (51.6)
Race, No. (%)		
Asian	32 (1.7)	7 (2.3)
Black	432 (23.5)	73 (23.9)
Caucasian	1120 (61.0)	213 (69.6)
Hispanic	30 (1.6)	0 (0)
Indian, American	5 (0.3)	4 (1.3)
Other or unknown	218 (11.9)	9 (2.9)
Bone density		
Type: Dual Femur, No. (%)	137 (7.5)	27 (8.8)
Bone Mass Density, mean (SD), g/cm^2^	0.538 (0.183)	0.926 (0.199)
Bone Density T-Score, mean (SD)	−1.154 (1.312)	−1.011 (1.438)
Type: forearm, No. (%)	92 (5.0)	7 (2.3)
Bone Mass Density, mean (SD), g/cm^2^	0.526 (0.252)	0.745 (0.221)
Bone Density T-Score, mean (SD)	−1.540 (1.768)	−1.629 (1.428)
Disease		
ICD-9, Distinct No.	3170	1891
Disease category, Distinct No.	723	442
Procedural		
No. (%)	559 (30.4)	97 (31.7)
CPT code, Distinct No.	202	39
Severe falls, No. (%)	297 (16.2)	33 (10.8)

The best result was obtained by using the following features: all the logistic regression classifiers from each view of data except procedural data, SVR classifiers from both forearm and dual femur bone density measures, and missing flags for procedural and both bone density measurements. The excluded features are SVM classifiers from demographics, disease, and procedural data, and logistic regression classifier from procedural data. Using this group of features, our model achieved an AUC of 0.713 (95% CI, 0.701–0.725) by bootstrapping the ten repeats of 10-fold cross-validation on the training set.

**Fig. 1:**
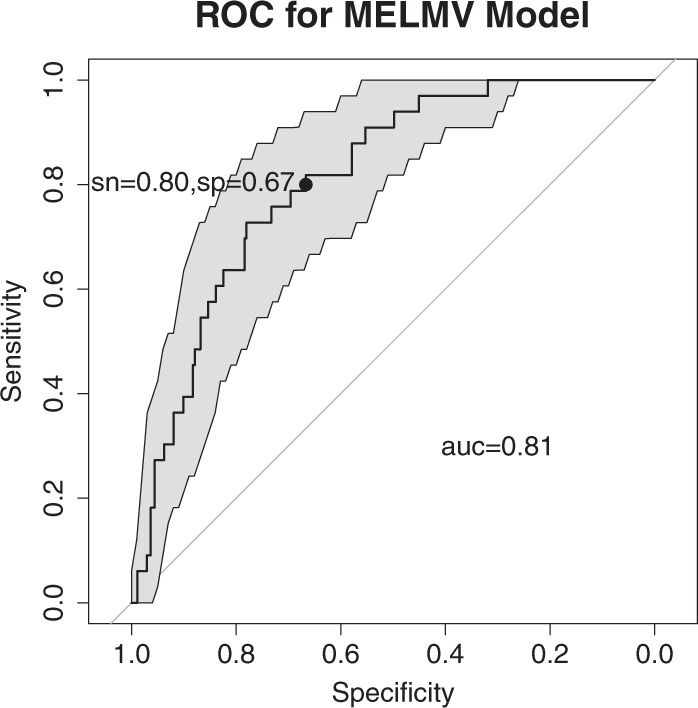
The performance of the multi-view ensemble learning with missing data classifier (MELMV) on the testing set. Model performance was evaluated by the receiver operating characteristic curve on a prospective testing set. The 95% CIs of specificity were also showed at shaded band. ROC: receiver operating characteristic curve; AUC: area under the receiver operating characteristic curve; sn: sensitivity; sp: specificity; MELMV: multi-view ensemble learning with missing value classifier.

The comparison of the performance with all the baseline models on the training and testing set is shown in Table 2. The AUC values of MELMV, LG, SVM, and random forest from the ten repeat of 10-fold cross-validation are 0.713, 0.668, 0.618, and 0.679, respectively. SVM’s AUC value is significantly worse on testing subsets than that of the other three models but it performs best on the training subsets, indicating that SVM more easily overfits data than the others. On the testing set, the MELMV model reached the best prediction performance with AUC 0.808 (95% CI, 0.740–0.876) with DeLong test, with a sensitivity of 80% specified, specificity is 67% (95% CI, 51–82%) (Fig. 1). The LG and SVM models reached 0.728 and 0.596 on the testing set. The random forest model reached an AUC of 0.753. This shows that our model-based imputation method has better contribution to model performance, and that our ensemble method performance is better than a baseline model like random forest. The MELMV model demonstrates more robust and better predictive power than both LG and SVM models.

**Table 2:** Model performance comparison with different imputation methods for missing data.

Modeling approach	Training set AUC (95% CI)	Testing set AUC (95% CI)
MELMV Model with Model-based Imputation	0.713 (0.701–0.725)	0.808 (0.740–0.876)
Random forest with Model-based Imputation	0.679 (0.664–0.695)	0.753 (0.665–0.841)
Model of ensemble of all classifiers	0.628 (0.616–0.640)	0.706 (0.622–0.790)
Single Logistic Regression Model with multivariable imputation	0.668 (0.653–0.682)	0.728 (0.726–0.730)
Single Support Vector Machine Model with multivariable imputation	0.618 (0.607–0.628)	0.596 (0.592–0.600)
Model of ensemble single view of LG and SVM	0.619 (0.605–0.634)	0.645 (0.643–0.648)

**Fig. 2:**
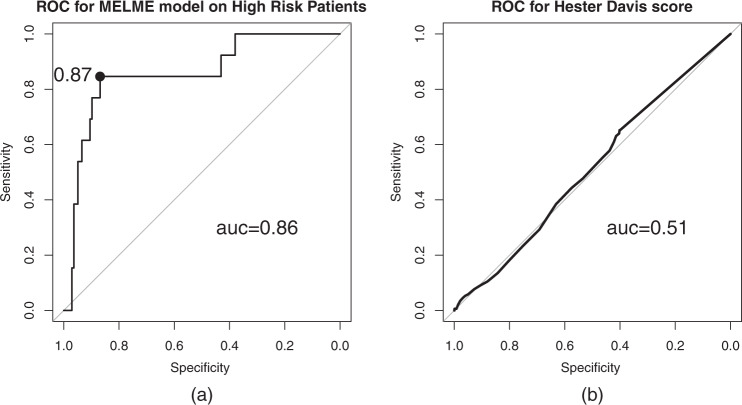
The comparison of the performance of the multi-view ensemble learning with missing data classifier (MELMV) and Hester Davis score on the high risk fall patients in the testing data. **a** Performance of the MELMV model evaluated by the receiver operating characteristic curve on the testing set with high risk of fall based on the Hester Davis score. **b** Performance of the Hester Davis score as severity fall classifier evaluated by the receiver operating characteristic curve on the testing set. ROC: receiver operating characteristic curve; AUC: area under the receiver operating characteristic curve; sn: sensitivity; sp: specificity; MELMV: multi-view ensemble learning with missing value classifier.

As our goal is predicting the severity of falls, our predictive model could be used on patients who have been identified as high-risk for falls. To test the performance on the group of high-risk fall patients, we used our prediction model only on the high-risk fall patients in the testing data. The high-risk patients were identified by their Hester Davis scores, those whose scores are higher than 10 are considered patients of moderate to high risk of fall. The AUC on the high-risk patients is 0.86 (95% CI, 0.731–0.989), which is 0.05 higher than the result on all the cases in testing set (Fig. 2). Additionally, although the Hester Davis score is not designed for predicting the severity of a fall, we want to verify if it also can predict severity of a fall beyond its designed goal. The right of Fig. 2 is the ROC curve of using the Hester Davis score as the classifier to predict the severity of a fall. We can see that the Hester Davis score has almost no predictive capability to determine the severity of a patient fall or fall resulting in a patient injury, even though it has been approved and commercialized as a method of assessment of patient fall risk. Therefore, it can be concluded that predicting risk of a fall and predicting the severity of a fall are two different issues. Our MELMV model used in addition to fall assessment tools like Hester Davis can reliably predict high-risk and high-severity patients, who should have the highest priority for fall prevention.

### The predictive performance of each view of data

For individual predictors in each data view, the ROC curve on the testing data is plotted in Supplementary Fig. 1. The AUCs demonstrated that diagnosis/disease type is the most important factor associated with the severity of patient falls. Other factors (demographics, bone density, and procedure) are also predictive. No single predictor could satisfactorily predict fall severity alone, using advanced ensemble learning will ensure that the final model has better performance than any single view model can obtain.

Studying the data in further detail, statistical tests indicated some differences between minor and severe injury levels with respect to certain variables. Advancing age predisposes patients to increased fall severity or injuries, as severe falls were more frequent in the elderly patient population. Bone density measurements indicate a trend, though not statistically significant (Supplementary Table 1). The race/ethnic group factor was noted to play a significant role in severe outcomes of falls (Supplementary Table 2). Additionally, in Table 3 we list the disease areas for which injury scores are significantly higher than the population mean, statistical power ≥80%, *p*-value < 0.05. Among the diseases with the highest injury score was occlusion and stenosis of precerebral arteries, which is not unexpected given the well-known consequences and effects of reduced cerebral perfusion to a patient’s level of consciousness.

**Table 3:** List of disease areas of the training dataset whose injury scores are significantly higher than the population mean, with statistical power ≥80%, *p*-value < 0.05.

*P*-value	Ave injury scores	No of cases	Disease area
0.01	5.30	10	Occlusion and Stenosis of multiple and bilateral precerebral
0.02	5.29	7	Rhinovirus Infection in conditions classified elsewhere
0.02	5.17	18	Venous (Peripheral) insufficiency, unspecified
0.00	5.02	46	Mixed acid-base balance disorder
0.01	4.91	33	Other diuretics causing adverse effects in therapeutic use
0.03	4.90	31	Hepatorenal Syndrome
0.04	4.89	35	Acute and chronic respiratory failure
0.04	4.88	24	Temporary tracheostomy
0.04	4.85	27	Kidney replaced by transplant
0.03	4.83	35	Hypovolemia
0.02	4.80	35	Cachexia
0.00	4.80	65	Cardiac pacemaker in situ
0.04	4.78	49	Encounter for palliative care
0.03	4.71	58	Closed (Endoscopic) biopsy of bronchus
0.03	4.70	54	Body mass index less than 19, adult
0.04	4.68	50	Occlusion and stenosis of carotid artery without mention of cerebral infarction
0.03	4.66	99	Other ascites
0.02	4.62	111	Other fluid overload
0.03	4.59	138	Long-Term (current) use of steroids

## Discussion

We developed and validated an advanced machine learning model based on multi-view ensemble classifier, which can be used to predict severe outcome or injury in all types of falls. The model employs multiple views of patient data encoding complementary information and integrates the predictive power of individual views together to achieve a robust predictive performance or score. To cater for pragmatic clinical usage, the MELMV model can perform well even in the presence of a rather significant amount of missing data values using model-based imputation. The multi-view learning process of MELMV limits the effect of missing data and will not affect whole samples, unlike other variable imputing methods. Hence, the MELMV model is more robust and generalizable than conventional single view learning models with variable imputing methods. In addition, the MELMV classifier model is easier to extend to include new views of data, as we just need to train sub-classifiers of the model on the new data instead of retraining the entire model from scratch. In summary, the new multi-view ensemble learning with missing data model is robust, generalizable, and extendable, as demonstrated in the comparison with other popular machine learning or statistical methods.

In addition, the MELMV classifier could be used immediately upon admission with as little information as age, sex, race, and diagnosis. The derivable advantage of severe fall prevention is dependent on how early the assessment is made, this is important in cases such as behavioral falls where patients intentionally fall. In practice, the MELMV tool could be employed in the clinical setting to predict severity after a fall assessment has been made using one of the aforementioned fall risk assessment tools such as Hester Davis, STRATIFY, etc. This extra layer of alert for the healthcare provider will allow for cost-effective implementation of more efficient and timely fall prevention strategies. The MELMV model is implemented in a web application, Severity Of Patient Falls Risk Assessment (SOFRA) to incorporate the severity risk score into the clinical workflow via the electronic medical record (EMR) to alert care providers (Supplementary Fig. 2). For the patients who have high risk for severe fall, we would like to advocate the intervention strategies should be strictly followed. They should be placed under very keenly focused, possibly round the clock, observation and monitoring including having more nursing resources dedicated to them.

There have been a few studies that attempted to use fall risk assessment tools for fall severity and injury prediction. Nilsson et al.^19^ made use of the fall risk assessment derived from the Downton Fall Risk Index (DFRI) to predict fall related injuries, fall related head injuries and hip fractures, and all-cause mortality in a large cohort of older patients. The research is a factor analysis that showed the DFRI score has significant relation to some kinds of injuries in the cohort of older patients, but there is no model performed to predict the severity of various kinds of injuries from falls in a broad spectrum of patients. Shinichi Toyabe^20^ also attempted to develop a risk assessment tool to predict severe injuries from falls using a combination of STRATIFY and Fracture Risk Assessment Tool (FRAXTM) scores. A model was developed that interprets a STRATIFY score of more than two and a FRAX score of more than ten as predictive for severe injury after falls. However, the severe injuries considered were only limited to bone fractures and intracranial hemorrhages as these, according to the authors, account for most severe injuries following falls.

Despite its advantages over existing tools and methods, the MELMV classifier method would need certain improvements. First, this study was conducted within Houston Methodist Hospital, the flagship hospital of Houston Methodist at Texas Medical Center. Our model needs validation of its generalizability across multiple hospitals. Our ongoing study includes conducting a validation of MELMV model cross the 8-hospital system of Houston Methodist. Second, the MELMV tool will be applied to predict high risk of fall injuries for the fall patients who have been identified by a commercial fall assessment tool. Third, for new patients with limited medical records, including first time admissions or transfers from other hospitals, especially in their first few hours of admission or transfer, little information is available to feed the model to predict the severity of falls. Nevertheless, as time progresses, the assessment of fall severity would be updated as more information, such as procedures and bone density scan results, becomes available.

In summary, we reported a powerful and generalized model using an integrative machine learning technique to predict the severity of all kinds of injuries of inpatient falls, and trained and validated the model in a cohort of over two thousand fall patients. Such a valuable tool fills the gap of one of the most vexing patient-safety problems facing hospitals today and will help to focus care givers on prescribing and implementing additional prevention or intervention strategies for those patients at high risk for injuries from severe falls, beyond standard-of-care intervention measures for all high risk fall patients.

## Methods

### Data source

There are approximately 600 inpatient fall incidents annually recorded in the Houston Methodist Hospital (HMH), the main campus of the 8-hospital system of Houston Methodist. For each recorded fall incident, the level of harm was documented using the Agency for Healthcare Research and Quality (AHRQ) Common Format Harm Score v.1.1 referenced in Patient Safety Network (PSN) with numbers from 1 to 9, Supplementary Table 3 describes the AHRQ harm score.^21^ Falls recorded with a harm level of 6 and above on this scale indicate significant harm and are therefore classified as severe falls. Using the Patient Safety Net (PSN) and Safety Intelligence system of HMH, we extracted reported inpatient fall events for the period of January 2011 to August 2015. The testing set was extracted from the period of May 2016 to December 2016. We then mapped those patients to our enterprise-wide clinical data warehouse, METEOR (Methodist Environment for Translational Enhancement and Outcomes Research), which integrates existing business data warehouse and patient records across the eight hospitals of the HMH system.^22^ The METEOR framework consists of two components: the enterprise clinical data warehouse (EDW) and a software intelligence and analytics (SIA) layer for enabling a wide range of clinical decision support systems to support clinical research and outcome studies. Using the METEOR clinical data warehouse, we retrospectively analyzed the fall patients’ medical records.

We collected the fall patients’ demographics data, which include sex, age, and race; all the admission diagnoses and chief complaints (International Classification of Diseases, Ninth Revision [ICD-9] code); all the procedural data within ten days of the falling date (CPT codes); and bone density measurements reported within one year. Any bone density measurement more than one year prior to the incident is not accurate enough to reflect the real bone density at the time of the fall. Bone density is extracted from the DEXA report (region, scan type, bone mass density, and T-score) using natural language processing (NLP).^23^ We preprocessed the diagnosis codes by keeping only codes that indicate injury or illness of higher hierarchy and ignoring those codes of lower hierarchy. Among falling patients, 8% had more than one fall incident. We treated each of these fall cases independently, without considering patient identity, as a patient’s data could vary at different fall dates.

This study was approved as a quality improvement investigation by the hospital administration and the Informatics oversight committee of the Houston Methodist system. This study has been reviewed by the Institutional Review Board (IRB) of Houston Methodist Hospital.

### Model development

Multi-view ensemble learning is a machine learning method that deploys multiple distinct feature sets to train multiple learning algorithms in order to obtain better predictive performance than any of the constituent learning algorithms alone. Multi-view ensemble learning has the potential to address high dimensional data. Research shows that multi-view ensemble learning improves the accuracy and generalization performance of the learning model. It has been used in learning areas, such as clustering, classification, and dimensionality reduction, etc.^24^ The method has also been applied successfully in a number of real world applications, including healthcare.^25–28^ Multi-view ensemble learning exploits each ‘view’ of the data, which comes from different resources and encodes different but complementary information for the whole ‘image’, learns each view of the data set with its own learning process, and finally constructs an ensemble of all the learning outputs from each view to obtain a single output as the final classification result with better predictive performance.

There are four views in our data set: patient demographics, disease diagnosis, bone density measures, and procedural data. They are distinct feature sets coming from different resources, reflecting different aspects of the patients’ information. Each view of the data in the training set was processed using two different base inducers, namely, a linear prediction model (logistic regression) and a nonlinear one (support vector machine), to compensate for each other. Both outputs are values between 0 and 1, indicating the predictive probability of a severe fall (Supplementary Fig. 3).

To handle the cases with missing data in some sets, we use a model-based machine learning method to impute the missing data.^29^ In general, the classifier is trained on available data of each view, and the ROC curve is calculated on the same data available. A threshold is used as an output for missing data, and a missing flag is created to indicate whether the output is a real predictive output from a real data sample or a threshold for missing data. The threshold is determined by the best cutoff on the ROC curve on available data, which maximizes the sum of the sensitivity and specificity. In this way, all the classifiers from different views of data sets have complete outputs for all samples, without being affected by distinct missing data conditions in each view (Supplementary Fig. 4).

In the ensemble step, we use the ensemble selection method to combine the component classifiers.^30^ That is, instead of using all of the component classifiers to construct an ensemble, a subset of classifiers will be selected to include in the ensemble to avoid overfitting, reduce redundancy of classifiers, and obtain the best performance of the final classifier. We also generalize the ensemble selection method in two ways. First, the missing flags are taken into account in the ensemble construction, not just the set of classifiers, as the missing flags contain the information of missing data, real output or not, which should be considered. The classifiers and missing flags together are considered as ‘features’ in the final model. Second, we use the wrapper feature selection method instead of heuristics to choose the optimal subset of classifiers and flags using logistic regression model.^31^ The best subset of features is selected to be included in the final ensemble model based on each subset’s predictive performance. The single output of the final model is a value between 0 and 1, indicating the predictive probability of a severe fall (severity index). More details of the structure of the multi-view ensemble model and the training of each individual view of data can be found in Supplementary Figs. 3 and 4.

### Model validation

The best subset of component classifiers and missing flags was settled using ten repeats of 10-fold cross-validation, and then the final model was fitted using the training set and tested in the separate testing set. To test the performance of the multi-view ensemble classifier model, we compared the model to single view logistic regression model and support vector machine (SVM) model. The single view model is meant to concatenate all views of the data as a single feature vector and then build a single model on all the features. The features in the model were optimized using ten repeats of 10-fold cross-validation. Multivariable Imputation method was used to deal with missing data in the training set before the two models were trained.^32^ Each run of cross-validation was trained with different imputing data to offset the effect of the randomness of the imputing data, and the final performance is estimated on the average performance of 10-fold cross-validation.

We evaluated whether the proposed ensemble method is better than prevalent machine learning methods for this application. With the same classifiers and missing flags as features, we trained a random forest model as the final model. Variables were selected in the random forest using 10 repeats of 10-fold cross-validation. We also compared with two simple ensemble models: ensemble of all the classifiers and missing flags, and the ensemble of single-view logical regression (LG) and SVM.

The area under the receiver operating characteristic curve (AUC) was calculated to measure the performance of the models. First, the average of AUC of the cross-validation repetitions on the training set was computed to estimate the overall performance of the MELMV model, LG, SVM, random forest and the other two ensemble models. Then, the performance of the models was evaluated on the testing set. We also tested our model on part of the testing set with high fall risk based on the Hester Davis scale deployed at Houston Methodist to assess whether the MELMV model offers more precise information than a commercial fall risk tool.

Analyses were performed using Python (Python Software Foundation) and R version 3.4.3 (R Foundation for Statistical Computing). The MySQL package in Python was used to automatically retrieve the training and testing data, and the liquidSVM^33^ and speedglm^34^ packages in R were used for generating the support vector machine (SVM) and logistic regression models. The ‘MICE’ package^35^ in R was used to impute the missing values both in the training data and testing data. pROC^36^ package in R was used for generating AUCs of the prediction results from the model. Reporting complies with The Strengthening the Reporting of Observational Studies in Epidemiology (STROBE) Statement.

### Statistical analysis

Model performance was evaluated based on the 95% CIs of AUC. The 95% CIs of the models on the training set was computed by Bootstrapping the 10 repeats of 10-fold cross-validation. In all, 95% CIs of the LG, SVM, and their ensemble model on the testing set was computed by bootstrapping 100 repeats on the testing set with different imputing data, DeLong test was used to compute the 95% CI of our model, random forest, and model of the ensemble of all features on the testing set.^37^ Model performance is statistically significantly better than another if its 95% CI exceeds the mean AUC of the other model.

### Reporting summary

Further information on research design is available in the Nature Research Reporting Summary linked to this article.

## Supplementary information

supplementary materials

reporting summary

## Data Availability

The datasets analyzed during the current study are not publicly available. Due to privacy and security concerns, the EHR data are not redistributable to researchers other than those engaged in this study.

## Code availability

Code for the algorithms used for analyzing the EHR data; development and evaluation the model; and statistical analysis are available from the corresponding author upon reasonable request.
