# Supplementary material for: Preventing inpatient falls with injuries using integrative machine learning prediction: a cohort study
Source: NPJ Digit Med. 2019 Dec 12;2:127. doi: 10.1038/s41746-019-0200-3 (PMC6908660; doi:10.1038/s41746-019-0200-3)
Supplement: Supplementary file 1 — supplementary materials [file 41746_2019_200_MOESM1_ESM.pdf]

Supplemental

Supplementary Figure 1. The ROC curves for classifiers of each view of data. The types of disease outperform other factors for predicting the fall severity.

Supplementary Figure 2. A screen shot of the SOFA webpage. A patient's severity risk score has been calculated and is shown at the bottom of the page after inputting the variables the model needed.

Supplementary Figure 3. The framework of the multi-view ensemble learning classifier for patient severe fall prediction.

Supplementary Figure 4. The structure of the unit of each view of data, comprising two base inducers: logistic regression and support vector machine, and missing flag.

Supplementary Table 1. ANOVA analysis result between minor and severe falls for training data on Age, Sex, and bone density.

Supplementary Table 2. Race/Ethnic Group Differences between minor and severe falls.

Supplementary Table 3. The meaning of the AHRQ Common Format Harm Score.

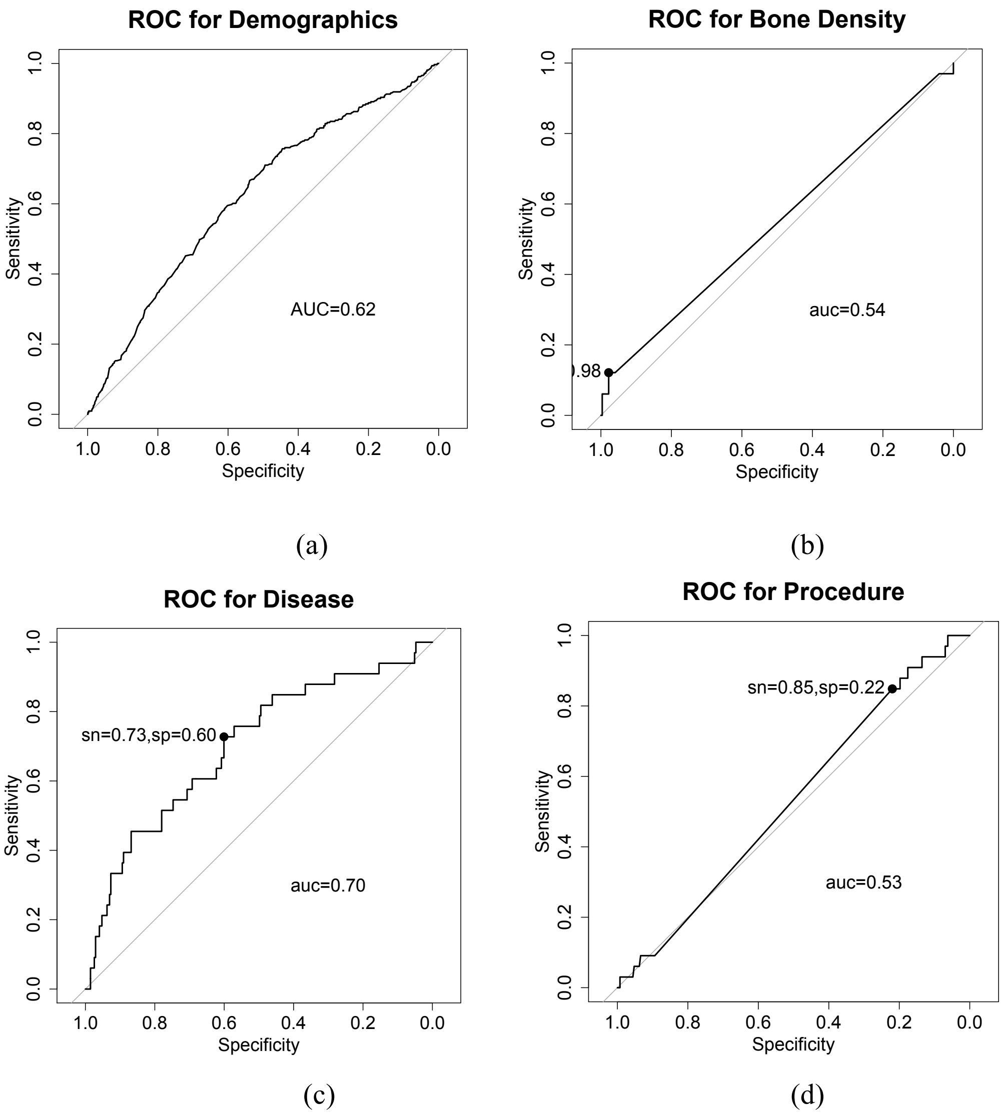

Supplementary Figure 1. The ROC curves for classifiers of each view of data. The types of disease outperform other factors for predicting the fall severity.

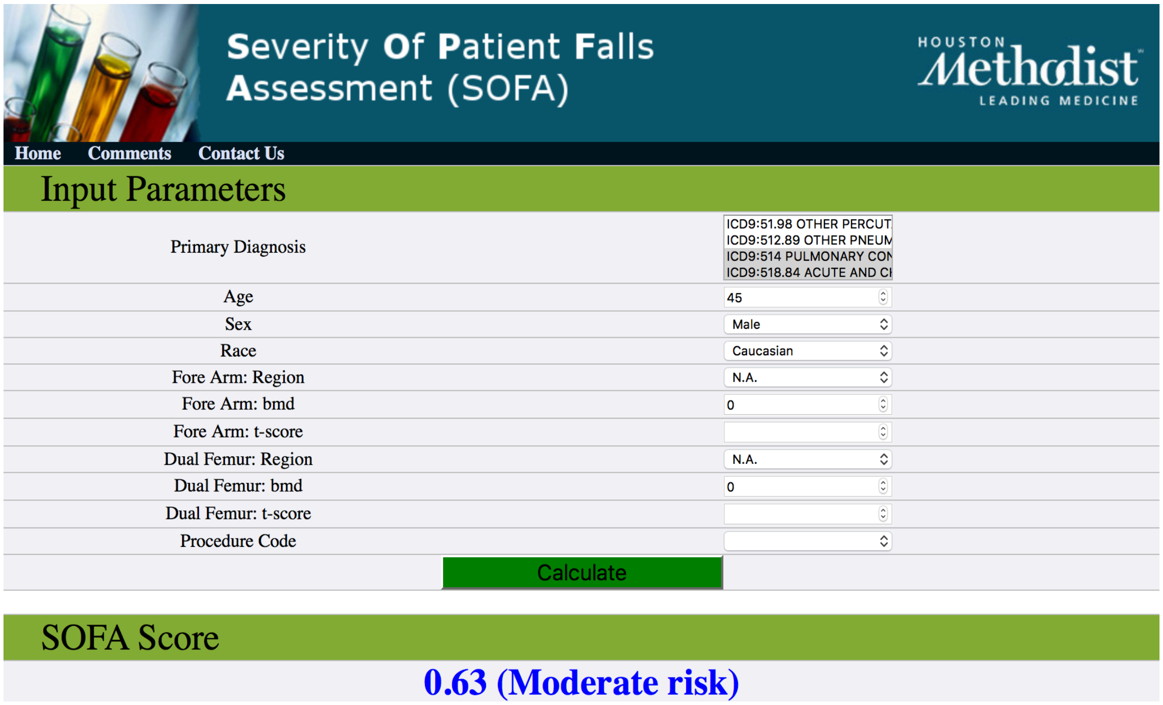

Supplementary Figure 2. A screen shot of the SOFA webpage. A patient's severity risk score has been calculated and is shown at the bottom of the page after inputting the variables the model needed.

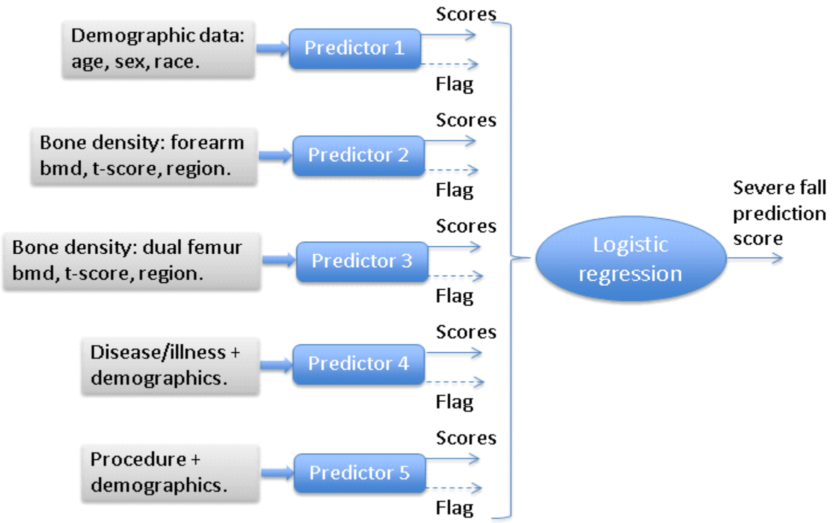

Supplementary Figure 3. The framework of the multi-view ensemble learning classifier for patient severe fall prediction.

The framework of the multi-view ensemble learning model is illustrated in **Figure 3**. The first level generates classifiers and missing flags from each view of data set. The inducers are SVR and logistic regressions. The predicted output borderline or threshold based on the ROC curve is assigned as the output for the missing data. An additional flag is used for the output to indicate whether the predicted score is an actual prediction or a borderline value. The second level combines all component classifiers' outputs and missing flags using a linear logistic model to construct an ensemble classifier. It should be noted that each patient might have multiple diseases or ICD codes, and each disease will generate one output from SVR and another from logistic regression. We chose the maximum outputs from multiple diseases to present the fall samples' classifier outputs from the disease view of data.

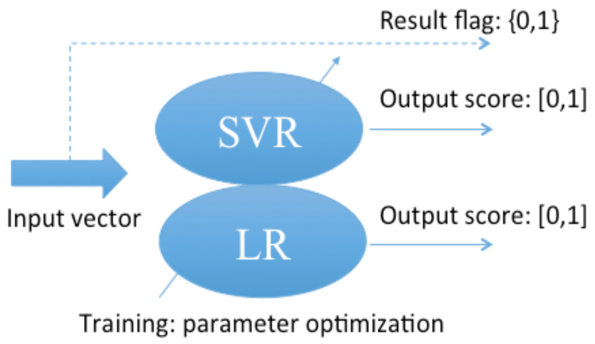

Supplementary Figure 4. The structure of the unit of each view of data, comprising two base inducers: logistic regression and support vector machine, and missing flag.

The unit for each view has a similar structure and is shown in **Figure 4**. The input of the classifier is a vector consisting of numerical or factorized variables of a data view. After training, the classifiers' outputs and missing flags are generated for the training data. When the flag is 0, the input data for that data view is not available and the output is the borderline based on the ROC curve on the training set. When the flag is 1, the output is the actual prediction from the input view of data.

Supplementary Table 1. ANOVA analysis result between minor and severe falls for training data on Age, Sex, and bone density.

	Severe Fall	Minor Fall	P-value
Age	67.2+/-16.1 (279)	62.4+/-15.7 (1413)	<0.0001
FA measure	0.16+/-0.04 (13)	0.145+/-0.05 (75)	0.188
DF measure	0.161+/-0.022 (20)	0.156+/-0.028 (108)	0.053
Sex	130/149 (Female/Male)	738/675 (Female/Male)	0.26

Supplementary Table 2. Race/Ethnic Group Differences between minor and severe falls.

	Asian	Black	Caucasian	Hispanic
Asian	-	0.04	0.43	0.60
Black		-	<0.0001	0.015
Caucasian			-	0.94
Hispanic				-

Supplementary Table 3. The meaning of the AHRQ Common Format Harm Score.

Harm score	Meaning
1	Unsafe Condition
2	Near Miss
3	No Harm
4	Emotional Distress
5	Additional Treatment
6	Temporary Harm
7	Permanent Harm
8	Severe Permanent Harm
9	Death
